# Inferring signalling networks from longitudinal data using sampling based approaches in the R-package 'ddepn'

**DOI:** 10.1186/1471-2105-12-291

**Published:** 2011-07-19

**Authors:** Christian Bender, Silvia vd Heyde, Frauke Henjes, Stefan Wiemann, Ulrike Korf, Tim Beißbarth

**Affiliations:** 1 German Cancer Research Center (DKFZ), Division of Molecular Genome Analysis, Im Neuenheimer Feld 280, 69120 Heidelberg, Germany; 2 University of Göttingen, Department of Medical Statistics, Humboldtallee 32, 37073 Göttingen, Germany

## Abstract

**Background:**

Network inference from high-throughput data has become an important means of current analysis of biological systems. For instance, in cancer research, the functional relationships of cancer related proteins, summarised into signalling networks are of central interest for the identification of pathways that influence tumour development. Cancer cell lines can be used as model systems to study the cellular response to drug treatments in a time-resolved way. Based on these kind of data, modelling approaches for the signalling relationships are needed, that allow to generate hypotheses on potential interference points in the networks.

**Results:**

We present the R-package 'ddepn' that implements our recent approach on network reconstruction from longitudinal data generated after external perturbation of network components. We extend our approach by two novel methods: a Markov Chain Monte Carlo method for sampling network structures with two edge types (activation and inhibition) and an extension of a prior model that penalises deviances from a given reference network while incorporating these two types of edges. Further, as alternative prior we include a model that learns signalling networks with the scale-free property.

**Conclusions:**

The package 'ddepn' is freely available on R-Forge and CRAN http://ddepn.r-forge.r-project.org, http://cran.r-project.org. It allows to conveniently perform network inference from longitudinal high-throughput data using two different sampling based network structure search algorithms.

## Background

Reconstruction of biological networks from data has become important in modern analysis of large data sets in genomics or proteomics. The aim is to infer pairwise regulations or influences of network nodes, such as genes or proteins, describing the system as a graph structure. With this graphical representation, the functional characteristics of a biological system can be visualised in a comprehensive and informative way. For this purpose, many approaches have been suggested in the past, including Boolean or Probabilistic Boolean Networks, Bayesian or Dynamic Bayesian Networks (DBN) or learning with differential equation systems and many more [1-5]. These methods rely on the measurement of network components, either under several experimental conditions or at different time points. The simultaneous measurement of time courses combined with different experimental conditions or even directed perturbation of components becomes an increasingly important way of characterising biological systems [6]. Corresponding modelling approaches try to describe the responses of model systems after external perturbation [7,8]. Recently, we proposed a framework that searches for optimal network structures by modelling the signal flow in a cell from an external stimulus to the downstream components and we showed how parts of a literature based reference network could be reconstructed [9]. The method is now implemented in the R-package *'ddepn' *which is available in the 'Comprehensive R Archive Network' (CRAN).

The purpose of this document is to give a comprehensive description of the package and its capabilities. Besides the original approach for network inference, three components are added to the package. First, as alternative to the Genetic Algorithm (GA) presented in our previous work, a Metropolis Hastings Markov Chain Monte Carlo (MCMC) sampling scheme is set up. This approach is based on a publication of Werhli et al. [10], but extended by the possibility to sample two types of edges directly (activation and inhibition). Hence, the package contains an optimisation algorithm (the GA) that is designed for converging to an optimal network, as well as the MCMC algorithm for true sampling of the space of possible network structures. Second, a prior probability model for the network structure is adapted from Fröhlich et al. [11], that can penalise differences of inferred edges to prior confidences of these edges. Again, we extended the previous model to include the possibility to model different edge types. Third, an alternative prior model is provided that models the scale-free characteristics of inferred networks, i.e. it tries to reconstruct networks with node degrees that follow a power law distribution. This approach was introduced before [12,13]. We describe how prior parameters can be adjusted to guide the reconstruction to be more or less close to the given reference and how reconstruction performance is affected by the prior parameter settings. Finally, advantages and disadvantages of both the GA and MCMC methods are discussed and a real data example is given that highlights how the prior knowledge inclusion is improving the outcome of the inference process.

## Implementation

The following sections provide a description of the different methods included in the package. The first section 'Network inference types' describes the network inference approaches. A short overview on the original method based on a GA is given in the first subsection 'Genetic Algorithm'. In section 'inhibMCMC' we present our novel approach for Markov Chain Monte Carlo sampling of network structures. The next section 'Prior knowledge incorporation' includes the two prior models for the inference. Subsection 'Laplace Prior' introduces our extension to the prior model of Fröhlich et al. [11], subsection 'Scale-Free prior' describes the implementation of the alternative prior model of Kamimura et al. [12].

In general, the networks to be inferred are encoded as directed (and possibly cyclic) graphs. Let *V *= {*v_i _*: *i *∈ 1 ... *N*} be the set of nodes representing the network components (proteins, genes, etc.) and Φ = *V *× *V *→ {0, 1, -1} an adjacency matrix defining a network. Each edge in Φ is defined as pair of nodes {*ϕ_ij _*: *i*, *j *∈ 1 ... *N *}, where 0 means no edge, 1 activation and -1 inhibition between two nodes. The networks are inferred using a data matrix *D *= {*d_itr _*: *i *∈ 1... *N*, *t *∈ 1 ... *T*, *r *∈ 1 ... *R*} holding the time-resolved data of *N *proteins in *T *time points, measured in *R *replicates each.

### Network inference types

#### Genetic Algorithm

In principle, a population of candidate networks is 'evolved' over a large number of iterations, starting with either a population of randomly drawn networks or by providing an initial population of networks. One can thus extract networks based on biological prior knowledge and feed them into the algorithm as a starting point for the network search. In each iteration of the algorithm, first up to a fraction 1 - *q *∈ [0; 1] of all candidate networks is selected that has a score larger than a given quantile of the scores of all current networks (we use the median). This is done to keep the best scoring networks in the population. Second, crossover is performed between pairs within a fraction *q *of the networks to allow exchange of parts of the networks. Third, mutation of a fraction of *m *∈ [0; 1] edges in each network is performed, changing an edge to one of the remaining states, e.g. if an activation edge is present, it can change to an inhibition or to no edge at the current position. This increases the chances to evade local optima and explore different parts of the search space, even if the local move is reducing the score. The details of the methods are described in our previous publication [9]. For our purposes, we set the parameters to *q *= 0.3 and *m *= 0.8 and recommend to use a population size of at least 500 networks to ensure broad sampling of the network search space.

#### inhibMCMC: Markov Chain Monte Carlo for two edge types

As an alternative to the GA, an MCMC structure learning approach to sample the space of possible networks is included. The sampler is based on a previous approach by Werhli et al. [10]. Because we allow to include two edge types for activation and inhibition in our networks, we change the MCMC sampler in the following way. Adding an edge is replaced by two moves, one for adding an activation and one for adding an inhibition. Further, we include a move for switching the edge type from activation to inhibition (and vice versa) as well as one type to simultaneously revert and change the type of an edge. This leaves us with six move types: *add activation*, *add inhibition*, *delete*, *revert*, *switch type *and *revswitch*. Inclusion of the novel move types is done to ensure that any edge can be changed to any other edge (w.r.t. to type and direction) in exactly one step. Using these move operations, for any given network structure all other structures can be constructed in a finite series of moves. Consider table 1 for an illustration of the edge transitions.

**Table 1:** Edge transitions and corresponding move operations.

	→	⊣	←	⊢	∅
→	-	*st*	*rev*	*rst*	*del*
⊣	*st*	-	*rst*	*rev*	*del*
←	*rev*	*rst*	-	*st*	*del*
⊢	*rst*	*rev*	*st*	-	*del*
∅	*addA*	*addI*	*addA*	*addI*	-

Possible edge transitions and the corresponding move to perform the transition. An edge is changed from the type shown in the first column to the type shown in the first row by the move shown in the corresponding table field. The following moves are possible: *addA*: Add activation, *addI*: Add inhibition, *del*: delete, *rev*: revert, *st*: switch type, *rst*: revert and switch type. The symbols for the edges are →: activation, ⊣: inhibition, ∅: no edge.

Now the essential relationships for the MCMC sampling procedure are repeated (as shown in Werhli et. al. [10]). The proposal probability of any network Φ_*k*+1 _that differs from a network Φ*_k _*by only one edge is:(1)

where *N*(Φ*_k_*) is the neighbourhood of a network *k*, i.e. all network structures that can be reached by a single edge operation. A move is accepted with acceptance probability(2)(3)

where the posterior distribution is(4)

 is a constant normalising factor that can be neglected for model comparison purposes. *P*(Φ*_k_*) represents the prior probability distribution for a network structure Φ*_k_*, which is described in the next section. The posterior *P*(Φ*_k_*|*D*) is the product of the prior *P*(Φ*_k_*) and the likelihood of the data given the network, defined in Bender et. al. [9] as:(5)

where *D *is the *N *× *T *× *R *data-matrix and  the optimized system state matrix, holding the active and passive states for each protein at each time. , ∀*i *∈ 1 ... *N *is the parameter matrix obtained during the HMM procedure from Bender et. al. [9], containing the parameter estimates for the Gaussian distributions for the active  and passive states . Details on parameter estimation as well as the system state matrix computation can be found in our previous publication [9].

We end this section by describing the determination of the neighbourhood  of a network. There are three cases to be considered to determine the cardinality of the neighbourhood of a network Φ*_k_*:

**I) addactivation/addinhibition ** (the number of node pairs that is not connected by an edge, where self-activations/inhibitions are not considered, w.l.o.g.)

**II) deletion/switchtype ** (the number of node pairs that are connected by an edge)

**III) revert/revswitch ** (the number of node pairs that are connected by an edge, and where the reverse edge is not already present)

Depending on the type of the move, the corresponding proposal probabilities *Q *can be calculated. Note that for Metropolis-Hastings MCMC structure sampling described here, the proposal distribution *Q*(Φ_*k*+1_|Φ*_k_*) can be non-symmetric, i.e. *Q*(Φ_*k*+1_|Φ*_k_*) ≢ *Q*(Φ*_k_*|Φ_*k*+1_) is allowed for any pair of networks Φ*_k _*and Φ_*k*+1_. To show that *Q *is not symmetric, we describe a counterexample for two simple networks with two nodes A and B (shown here as adjacency matrix:):

Φ_2 _is reached by adding the edge *A *→ *B*, and going back from Φ_2 _to Φ_1 _is done by deleting this edge. The number of neighbours of the first network is calculated as follows. According to I), 2 neighbouring networks can be reached by adding a single activation edge, as well as 2 networks by adding a single inhibition. Following II) and III), there are no edges that could be deleted, reverted or whose type could be switched. In total, there are 4 neighbouring networks to Φ_1_. Analogous to that, for the second network there is one reachable neighbour by adding an activation and one by adding an inhibition, one edge can be deleted, and one reverted, and for one edge a type switch as well as a reverse-type-switch is possible, totalling in a neighbourhood of 6 networks for network Φ_2_. Thus, the proposal distribution is not symmetric for all possible networks.

### Prior knowledge incorporation

#### Laplace prior

Based on the structure prior of Fröhlich et al. [11], a prior model is proposed that also incorporates different types of edges and a more fine-grained control of the prior influence. Networks are encoded as mentioned above. We need a matrix *B *= *V *× *V *→ [-1, 1] containing prior confidences for each edge, which can be obtained in various ways. Here, one example is given how to derive *B *using the KEGG database [14]. The approach is similar to the one described by Werhli et al. [10], but preserves the information on the type of the edges.

First, we download the signalling and disease related networks from KEGG (see the documentation of the data set *kegggraphs *in the package for a list of all pathways). The number of occurrences of each node pair *v_i _*and *v_j _*in all pathways is counted and recorded in a matrix *M *= *V *× *V *→ ℕ. Further, it is counted how often each node pair is connected via an activation or inhibition edge in all reference networks and the corresponding numbers are recorded in two matrices *M_act _*and *M_inh_*, both with the same dimensions as *M*. Note that for pairs of nodes that do not occur in any reference network (i.e. *M_ij _*is 0), we set the confidence score to 0. The prior confidence matrix *B *is thus defined as:

assuming that the type of each edge is consistent in all reference networks. This leaves positive confidences for activation edges and negative confidences for inhibiting edges. The larger the absolute value of the confidence score, the stronger is the belief in the presence of this edge.

No matter how *B *was derived, to calculate the prior belief for a network structure Φ we assume all edge probabilities to be independent:(6)

We calculate the difference between an edge in the inferred network Φ and the prior *B *and include a weight exponent *γ *∈ ℝ^+^to obtain the weighted difference term:(7)

The prior belief for an edge in the network is then modelled as(8)

which penalises large differences from the network structure Φ to the prior belief *B*.

Now we derive upper and lower bounds for the prior influence, in the general case for two edge types. Let *λ*, *γ *∈ ℝ^+^. If the edge type information is used, all differences Δ*_ij _*lie in the interval [0; 2*^γ^*], because *ϕ_ij _*∈ {0,1, -1} and *b_ij _*∈ [-1; 1]. Without edge type information, we ignore the signs in both Φ and *B*, leading to Δ*_ij _*∈ [0; 1*^γ^*], because *ϕ_ij _*∈ {0, 1} and *b_ij _*∈ [0; 1]. Because the bounds for *P *(Φ) will not change in either case, only the case for including edge type information is shown in the following.

For the moment, let *γ *= 1 and consider the limits of the exponential term in equation 8:

This means that(9)

**Figure 1:**
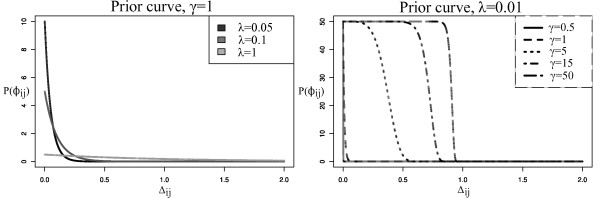
**Laplace prior curve**. Unnormalised prior densities, depending on difference Δ*_ij_*. Left: *γ *is constant, for increasing *λ *a 'flattening' of the prior curve can be observed. For small *λ*, small differences to the reference retrieve higher weight than large differences. For large *λ*, all differences are weighted approximately equal. Right: *λ *is fixed, when *γ *increases, a plateau at the upper bound  can be seen. This means that small differences to the reference are not penalised as strong as for small *γ*, leaving the control that up to some deviance from the reference a high prior weight is retained.

Since Δ*_ij _*≥ 0, ∀*γ *∈ ℝ^+^, the bounds are valid for *γ *∈ ℝ^+^, too. Figure 1 shows on the left side the prior curve for equation 8 when λ ∈ {0.05, 0.1, 1} and *γ *= 1. As it can be seen there, with increasing λ the (unnormalised) prior probability curve flattens out, giving unbiased probabilities for each value of Δ*_ij_*. The maximum value is bounded by . On the right side of Figure 1 we set *λ *= 0.01 and increase γ ∈ {0.5, 1, 5, 15, 50}. This results in a broader prior probability plateau at the upper bound for small differences Δ*_ij_*, suggesting that *γ *can be used to control how strong small differences of inferred edges to their prior confidence should be penalised. Extending the plateau of high prior probability will lead to high prior weights for edges with absolute confidence values not equal to 1, and additionally will leave a strong penalisation of larger differences.

**Figure 2:**
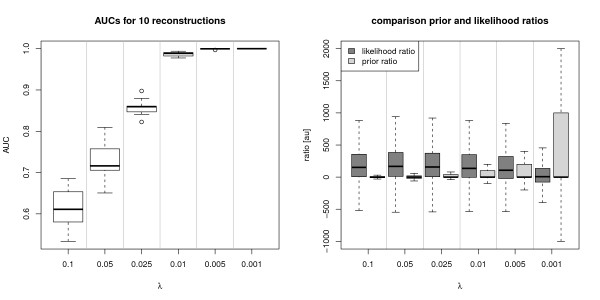
**Prior influence on reconstruction performance of inhibMCMC**. Diagnostics for inhibMCMC of a randomly sampled network (N = 15), 50000 iterations, burn-in 5000, *γ *= 1, varying *λ*. The sampled network was used as prior confidence, i.e. the prior knowledge was 'perfect' in this test. Left: The smaller *λ*, the stronger the prior influence was and the closer the inferred networks were to the prior (reflected in increasing AUCs). Right: Comparison of Likelihood and Prior ratios, depending on *λ*. *λ *should be chosen such that the prior and likelihood ratios vary in a comparable range. For instance, based on the plot, set *λ *= 0.005.

The prior parameter *λ *should be adjusted in a way that it exceeds the changes introduced by the likelihood, if strong bias towards prior knowledge is desired during inference. For inhibMCMC, we suggest to inspect the likelihood and prior ratios for various settings of *λ *and choose *λ *in a way that both ratios are approximately equal (see also Figure 2). To do this, transform equation 8 to log scale:

Now consider the prior and likelihood ratios on log scale, i.e. the differences of the log priors and log likelihoods. To make the prior capable of having substantial influence on the inference, the log prior differences should be on similar scale as the log likelihood differences. For instance, if the log likelihood differences are on the scale of 10^3^, set *λ *= 10^-3 ^and *γ *= 1, such that  will be in the range of the thousands. The first part of the prior (-*log*(2) - *log*(*λ*)) cancels out in the difference and does not have an influence. This means, that the prior influence is controlled over the second part, which is zero for no difference to the prior and and can become very large for differences *>*0. Hence, edge mismatches between the reference and the inferred net guide the structure search and the strength of the influence can be controlled using different settings of *λ*.

**Figure 3:**
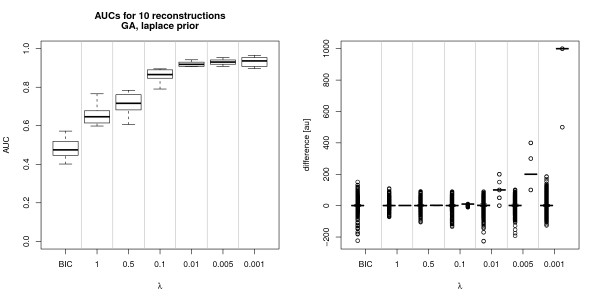
**Prior influence on reconstruction performance of GA**. Results for GA reconstruction for one sampled network (N = 15), population size p = 500, number of iterations 1000, crossover/selection rate q = 0.3, mutation rate m = 0.8, *γ *= 1. As in figure 2, the sampled network was used as 'perfect prior knowledge'. Left: AUC values without prior (column BIC) and for various settings of *λ*. When *λ *was decreased, the AUCs increased. However, unlike the inhibMCMC example, AUCs did not approach a value of 1, giving evidence that the GA converges to a local optimum. AUCs for BIC score optimisation were bad, emphasising the need for prior knowledge inclusion for larger networks. Right: Likelihood and prior differences. Since most of the observed prior differences were zero, only the non-zero values are shown. For each setting of *λ*, the left box corresponds to the observed distribution of likelihood differences, the right box to the prior differences. In the BIC column, only the likelihood difference distribution is shown, since no prior was used in this case.

For the GA, adjusting *λ *is slightly different. Instead of tracking the log likelihood and log prior differences between subsequent networks in the Markov Chain, the unlogged likelihood and prior differences (i.e. the absolute differences in the likelihood and prior, rather than their ratios) for each changed individual to a given population quantile (we usually use the median) have to be recorded. In the first iterations of the GA, the individuals in the population will be widely spread around the network structure search space and changes in the likelihoods and priors will be rather large. Once the population approximates an optimum, the changes will become smaller and be centred around zero. A strong prior will assure faster convergence to an optimum that is close to the reference. However, giving a guideline on how to set *λ *for the GA is difficult, because already rather 'weak' prior strength (in terms of the settings for inhibMCMC) seems to have a substantial influence (see Figure 3) and the true impact of the prior might vary in different data sets. It seems reasonable to find some *λ *that gives average prior differences slightly above zero, which means that the final score, i.e. posterior, is biased towards the prior confidences on average. In general, finding the right choice for the prior parameters is not trivial. We suggest to use the above rationale to find an initial estimate and to iteratively update and improve the settings. This requires evaluation of the results obtained using a specific setting for the prior involving expert knowledge on the field studied. Depending on that, subsequent modifications to the initial guesses might be necessary and the reconstruction has to be repeated.

#### Scale-free prior

A different way of specifying a prior model was introduced by Kamimura et al. [12], as well as by Sheridan et. al. [13]. It is assumed that the networks have a scale-free architecture and that the degree of a node follows a power-law *P*(*d*) ∝ *d*^-*γ*^, where *d *is the number of edges adjacent to a node. For any graph structure Φ with fixed number of nodes *N*, a prior probability can be calculated as follows. First, assign a probability *P_i _*to each node *i *∈ 1... *N*:

This probability decreases when *i *gets large, and all *P_i _*sum up to 1, i.e. . *μ *is in the range 0 *< μ <*1 and defined as , *γ *∈ [2; ∞[.

Assuming independent node selection proportional to *P_i_*, and introducing a parameter *K *that controls the mean number of edges, the probability of two nodes not being connected is defined as

The probability of any structure Φ*_σ _*= (*V*, *E*) of node set *V*, edge set *E *and a permutation *σ *= {*σ*_1_, ..., *σ_N_*} of all nodes in Φ is then

A number of *B *permutations *σ *are generated, resulting in one graph Φ*_σ _*for each permutation. The final probability of Φ is averaged over the prior probabilities of all permutations networks:

For a detailed description of the model we refer to the previous publications [12,13,15]. The scale-free prior can be used in cases where no information on edge confidences is available. During inference with the scale-free prior model, sparse network structures will be preferred, because high node degrees are penalised by the prior model. In our implementation the scale-free model can be selected by passing the argument *priortype="scalefree" *to the function call *ddepn*. Further the arguments *gam*, *it *and *K *have to be provided. Kamimura et al. give hints on the proper setting of the arguments. In the following sections, we assume that prior edge confidences are present and suggest the use of the Laplace prior. However, the scale-free prior might be a reasonable substitute for modelling more general characteristics of network structures and thus interesting for further analyses.

## Results and Discussion

### Testing the Laplace prior influence

For both the GA and inhibMCMC sampler several tests were performed. The aim was to show that the inference could be influenced in a way that on the one hand the result was close to a given reference network and on the other hand allowed to confute the prior, when evidence from the data got strong enough. The following rationale was applied for these tests. First, we assumed that our prior information was true. To ensure this, a network with *N *= 15 nodes was sampled, data was generated depending on this network structure and the original sampled network was used as Laplace prior matrix *B*. Sampling of the network and data were described previously (see Bender et. al. [9]). Both the prior confidences and inferred edges only take on values ∈ {0, 1, -1}, so the absolute differences were either 0, 1 or 2. All differences larger than 0 should have been strongly penalised, so we set *γ *= 1, leading to a sharp decrease of the prior density (equation 8) for Δ*_ij _>*0. Each mismatch in an inferred edge to the prior was thus given a weight close to 0 (see Figure 1). We performed tests of the reconstruction performance for the following cases:

**GA, BIC score optimisation **1000 iterations, *p *= 500, *q *= 0.3, *m *= 0.8 no prior

**GA, Laplace prior **1000 iterations, *p *= 500, *q *= 0.3, *m *= 0.8, *γ *= 1, *λ *∈ {1, 0.5, 0.1, 0.05, 0.025, 0.01, 0.005, 0.001}

**inhibMCMC, Laplace prior **50000 iterations, burn-in 5000 iterations, *γ *= 1, *λ *∈ {0.1, 0.05, 0.025, 0.01, 0.005, 0.001},

We performed *n *= 10 independent reconstructions on the same original network and data for both the GA and inhibMCMC samplers and calculated the Area Under Curve (AUC) of the Receiver Operator Characteristic (ROC) curves for each sampling run to measure the quality of the inference. AUCs were calculated as follows. For inhibMCMC, 50000 iterations were performed in each run with a burn-in phase of 5000 iterations. Final networks were generated by including an edge into the network that was present in at least a given proportion *th *∈ [0; 1] of the 45000 non burn-in networks. By varying *th *between 0 and 1, for each setting of *th *the number of true positive, false positive, true negative and false negative edges of the final network compared to the original network could be counted. ROC curves were set up and the AUCs calculated as area under the ROC curves.

Figure 2 shows the AUC scores for the inhibMCMC test. It can be seen that for decreasing *λ *the reconstruction performance increased. For *λ *= 0.005 and *λ *= 0.001 the reference network could be successfully reconstructed (AUCs around 1). We suggested to inspect the observed likelihood ratios during the sampling runs and set *λ *such that the quotient  is on a comparable scale (see section Laplace Prior). The right plot of Figure 2 shows the likelihood and prior ratios for one inhibMCMC run. For *λ *= 0.001 the prior ratios varied over a much broader range than the likelihood ratios, which lead to inferred networks that were nearly identical to the prior network, as it can be seen in the AUC of around 1. For increasing *λ *the likelihood ratios showed a larger variance than the prior ratios, which lead to decreasing AUCs and more variable inferred networks in turn. Thus, the setting of the prior parameters determines how robust the reconstruction of the networks is. The settings have to be carefully adjusted to preserve robustness, but leave enough variance to gain additional knowledge, represented in the data, too. The test for the GA reconstruction is shown in Figure 3. AUCs for the GA were calculated similar than for inhibMCMC, where final networks were estimated by including edges if they appeared in at least a fraction *th *of the networks of the final population. On the left hand side of Figure 3 the AUC distributions are shown. Using the BIC score optimisation for the reconstruction of a network of size N = 15, it is apparent that the performance of the GA drops significantly, with AUCs around 0.5 compared to the case for N = 10 in Bender et. al. [9], where performance was still good with AUCs around 0.73. This strong decrease of performance with increasing number of nodes emphasises the need for the inclusion of prior knowledge to produce reliable results, especially when the network size is increasing. When using prior knowledge, for decreasing *λ*, also an increase in the reconstruction performance was observed. For *λ *= 1, the performance of the GA was comparable to inhibMCMC performance with *λ *= 0.1, the improvement in reconstruction performance can be controlled similar to the case for inhibMCMC using smaller settings for *λ*. Using *λ *≤ 0.01 gave comparable reconstruction results with AUCs above 0.95, but in contrast to inhibMCMC, the reference network could not be inferred entirely for even smaller settings for *λ*. The GA seems to reach a local optimum, but does not find the true network, even for the test situation where the prior strength is increased subsequently.

On the right hand side of Figure 3, the likelihood and prior differences are shown. As stated in section 'Laplace prior', *λ *should be set such that the prior differences are slightly above zero. As depicted in Figure 3, setting *λ *= 0.1 lead to prior differences of around 10 and already had a strong influence on the reconstruction performance and could be used as appropriate setting for *λ*. Nevertheless, it remains difficult to find the proper *λ*, and it is ongoing work to find reasonable ways of identifying 'good' parameter settings for both inhibMCMC and the GA.

### Note on the choice of the algorithm

The question, of course, arises, which algorithm to choose. One should be aware, that the purpose of both approaches differs. The GA is used for performing optimisation of the network structure, while inhibMCMC explicitly samples the space of networks. However, both methods can be used to generate final estimates of the network structure and provide the user with a confidence of each edge in the final inferred network. The influence of the prior seems to be less controllable in the case of the GA, since rather 'weak' prior settings (compared to the inhibMCMC case) already had a strong impact, but increasing the prior strength always left some errors in the reconstructed networks. However, a clear dependency on the prior strength could be observed in both cases and a rough guideline for finding a suitable setting for the prior hyperparameters could be given. In general, finding the trade-off between strength of the prior and the influence of the data is of central importance. Too strong prior influence will only reproduce the prior knowledge and not allow for novel insights from the data. If the prior is too weak, the inference might not be able to identify the underlying network structure, due to e.g. too wide time intervals during the measurements, noise in the data, or nodes that were not measured at all.

As a last point, we consider the computational demands of both approaches. Clearly, the GA is much more expensive in terms of computation time. As an example, consider reconstruction of networks with the following settings (as we currently are using them): population size *p *= 500, *iterations *= 1000, *q *= 0.3, *m *= 0.8. In each iteration q * *p *= 150 individuals are processed in the crossover step and m * *p *= 400 individuals are processed during the mutation step. For each of these 150 + 400 = 550 operations, the time limiting step is the Viterbi Training algorithm including Hidden Markov Model (HMM) computations. In our experience, for networks of size around *N *= 15, Viterbi Training is computed in less than one second, leading to an estimate of total computation time of (1000 * 550) seconds, corresponding to *~ *6 days. For inhibMCMC, we usually use 50000 iterations for one sampling run, which means 50000 times the Viterbi Training in each sampling. This corresponds to approximately half a day for one network reconstruction, meaning that more than 10 independent samplings can be performed in the same time as needed for one reconstruction with a GA. If parallel computing architecture is available, the GA computation time can be reduced to a few days, but also the independent inhibMCMC runs can be distributed on different computing cores or nodes, making multiple parallel network reconstructions possible in about half a day. So due to the computational burden of the GA and the improved control of the prior strength in inhibMCMC, it is suggested to prefer inhibMCMC over the GA.

### RPPA data from breast cancer cell line HCC1954

**Figure 4:**
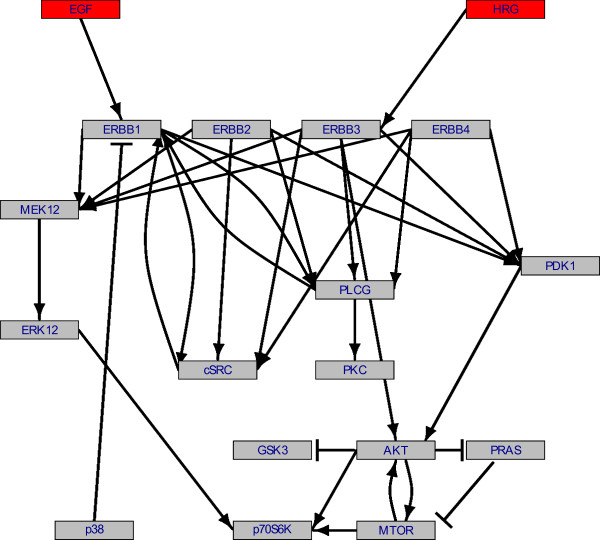
**Prior network**. Prior network derived in discussion with our lab staff after setting up an edge confidence matrix using the KEGG database.

To demonstrate how the prior knowledge inclusion improves reconstruction results from real data, we show an inference performed on a series of protein phosphorylation measurements for proteins selected from the ERBB signalling network. Measurements were generated on Reverse Phase Protein Arrays (RPPA, [16]) from HCC1954 cells after ligand stimulation with EGF, HRG and the combination of both. The data set is attached to our R-package as dataset *hcc1954 *and described in our previous publication [9]. Prior edge confidences were generated as described in section 'Laplace Prior', and a final reference network was assembled as follows. Edges with confidence ≥ 0.1 were included in the prior network, while the edge type information was preserved. This threshold fits best our expectations on the prior network. Additionally, several edges were included manually, that were described in current literature resources. The prior network is shown in Figure 4.

**Figure 5:**
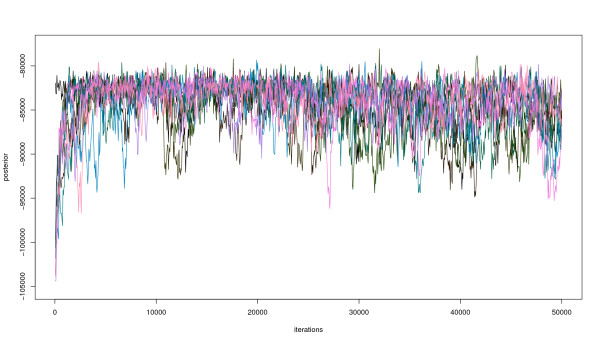
**inhibMCMC posterior traces for 10 MCMC chains**. Posterior traces of 10 inhibMCMC chains, 50000 iterations, *λ *= 0.0025, *γ *= 1 and a thinning interval of 50, showing the convergence to the stationary distribution after several thousand iterations.

Using this prior network, we applied inhibMCMC inference with 50000 iterations, where the first 25000 iterations were regarded as burn-in and discarded. The following parameters were chosen: *λ *= 0.0025, *γ *= 1. To assess convergence and ensure robustness of the results, 10 independent inhibMCMC chains were run in parallel, each starting at a randomly sampled initial network structure. Figure 5 shows the posterior traces of the 10 MCMC chains. It can be seen that the posterior probabilities converge to a stationary distribution after several thousand iterations. Comparing the number of activation and inhibition edges sampled in each of the 10 runs (after the burn-in phase), it can be seen that similar numbers are found, hinting at good mixing properties of the sampler (compare additional file 1). Note that each chain might visit distinct networks with similar posterior during the inference. According to the posterior probability, the chains seem to be converged, but there might be still substantial differences in the high scoring networks, reflected in increased variation in the numbers of edges. For instance, panel MTOR, column CSRC in supplementary figure S1 (in additional file 1) shows rather high variation in the number of activating and inhibiting edges. Nevertheless, the shift in the number of sampled edges (higher number of inhibitions compared to activations in the example) reflects stronger support for the inhibition in the data and might be of interest for further investigation of this particular edge. It is difficult to overcome this problem, but we think that using a suitable summarisation method to identify edges (e.g. as shown below) can help to identify true consensus networks described in the different inhibMCMC chains. However, the user should be aware of this issue and take precaution to avoid misleading inference results. We also refer to Cowles and Carlin [17] for a good review on convergence in MCMC methods.

For each run, a summarised network was generated by including a particular edge, if it occurred in at least a fraction *th *∈ [0; 1] of the 25000 non burn-in networks. This left us with 10 summarised networks, that were merged into a consensus network. For this, we counted, how often each edge was an activation, inhibition or empty edge in all of the 10 summarised networks. A simple majority rule decided on the final type of the edge. In the case of ties, the edge type was chosen that had the larger posterior probability average over all summary networks with the same edge type.

**Figure 6:**
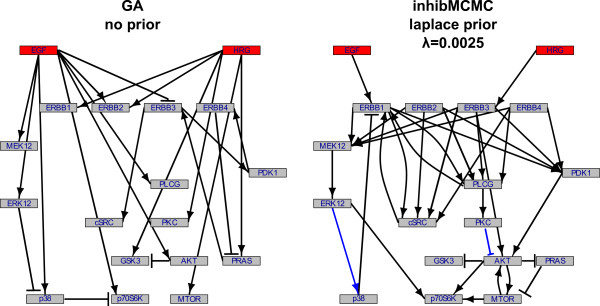
**Comparison of network reconstruction without and with prior knowledge incorporation for data set hcc1954**. Comparison of the network inferred using the GA and BIC score optimisation (left, adapted from Bender et. al. [9]) and using inhibMCMC together with the Laplace prior, *λ *= 0.0025 (right). Using the prior results in correct identification of signalling cascades like MAPK (EGF→ERBB1→MEK12→ERK12→p70S6K) or PI3K/AKT (HRG→ERBB3→PDK1→AKT). Additionally, two new edges were found, marked in blue (ERK12→p38 and PKC⊣AKT). These reflect changes in the connectivity that were strongly supported by the data and that were inferred although not included in the reference.

The following rationale was applied for inferring a network from the HCC1954-data using our assembled prior network. Because the ERBB network has already been extensively studied, we assume that much of this information is true. We intended to include a strict bias towards the a priori known edges, guaranteed by the prior hyperparameter *λ *set to 0.0025. By this, the general ERBB scaffold is retained unless there is strong support included in the data that contradicts the prior knowledge. The question is how to set the inclusion threshold used to determine the edges that are contained in the final network. Under our settings, for values *th *∈ [0.7, 0.85], the inferred network equals the prior network, and no new information can be gained. For higher inclusion thresholds *th >*0.85, edges are disappearing subsequently. This is expected because in the sampling procedure it is unlikely that an edge is contained in very high proportions of all sampled networks. Indeed, in our samplings at *th *= 0.94, no edges remain in the final networks. Therefore we decided to decrease the inclusion threshold until differences to the prior were observed. This was the case at *th *= 0.69. The inferred network using prior knowledge for this setting of *th *is shown in Figure 6 on the right side. The left side of Figure 6 displays the inferred network, when the GA and BIC score optimisation was used. It is apparent that the inference was improved using the prior knowledge when looking at the structure of known signalling cascades. For example, the MAPK kinase cascade EGF → ERBB1 → MEK12 → ERK12 → p70S6K or the cascade HRG → ERBB3 → PDK1 → AKT were inferred, which could be expected, because these are major signalling cascades that are ubiquitously present in biological systems. Using this prior setting, the inferred network is strongly biased towards the reference network, and only two new edges could be seen in Figure 6 (marked as blue edges): ERK12 → p38 and PKC ⊣ AKT. To allow more differences in the network structure, the parameter *λ *can be increased. The purpose here is to pinpoint the way of how bias towards the reference can be controlled using the *λ *parameter. For a detailed analysis of networks, e.g. under different experimental conditions, a suitable *λ *should be identified first by comparing inference results to the expectations on the network structures. Once a setting for *λ *is determined, the resulting network structures and inferred model parameters (see additional file 2 for an example plot of the model parameters) can be further investigated.

## Conclusions

We present our R-package 'ddepn' for inference of signalling networks from longitudinal high-throughput data. The method is able to model the effects of external perturbation, as it might be introduced by external stimulations or inhibitions. Two different network structure search algorithms are available in the package, a GA performing network structure optimisation and a Metropolis Hastings MCMC approach that samples the space of possible networks. We extend MCMC structure sampling by the ability to sample two edge types, one for activation and one for inhibition. Further, two models for the inclusion of prior knowledge are included in the package. The first uses a reference network as guidance for the inference (Laplace prior), the second uses a general property of biological networks, namely that node degrees follow a power law and high node degrees are penalised (scale-free prior). We also give a guideline on how to adjust parameters for the Laplace prior model, such that precise control on how close the reconstruction will be to the prior knowledge is possible. We show the dependence of the reconstruction performance on the prior parameter setting and give an assessment of both methods and their usage. Finally, for a data set measuring phosphorylation of proteins related to the ERBB signalling network, it is described, how inclusion of the prior is improving the outcome of the network reconstruction.

## Availability and requirements

**Project home page **http://ddepn.r-forge.r-project.org

**Operating systems **Linux, Windows

**Programming language **R

**Other requirements **graphviz

**Licence **GNU GPL

## List of abbreviations

**AUC**: Area under curve; **CRAN**: Comprehensive R Archive Network; **GA**: Genetic Algorithm; **HMM**: Hidden Markov Model; **MCMC**: Markov Chain Monte Carlo; **ROC**: Receiver Operator Characteristic; **RPPA**: Reverse Phase Protein Array.

## Authors' contributions

Concept and computations, as well as writing the manuscript were done by CB. SvdH ran several GA reconstructions. FH performed the RPPA experiments, and together with UK helped with the design of the prior network. TB gave advice on experiment design and writing the manuscript. SW proofread the manuscript. All authors read and approved the final manuscript.

## Supplementary Material

Additional file 1 **Edge confidences across 10 MCMC runs**. Shows the confidences for each edge obtained in multiple inhibMCMC runs. Click here for file

Additional file 2 **Model parameters for Gaussian distributions**. Shows the Gaussian model parameters for active/passive states of each protein. Click here for file
