# Supplementary material for: Inferring signalling networks from longitudinal data using sampling based approaches in the R-package 'ddepn'
Source: BMC Bioinformatics. 2011 Jul 19;12:291. doi: 10.1186/1471-2105-12-291 (PMC3146886; doi:10.1186/1471-2105-12-291)
Supplement: Additional file 1 — Edge confidences across 10 MCMC runs. Shows the confidences for each edge obtained in multiple inhibMCMC runs. [file 1471-2105-12-291-S1.PDF]

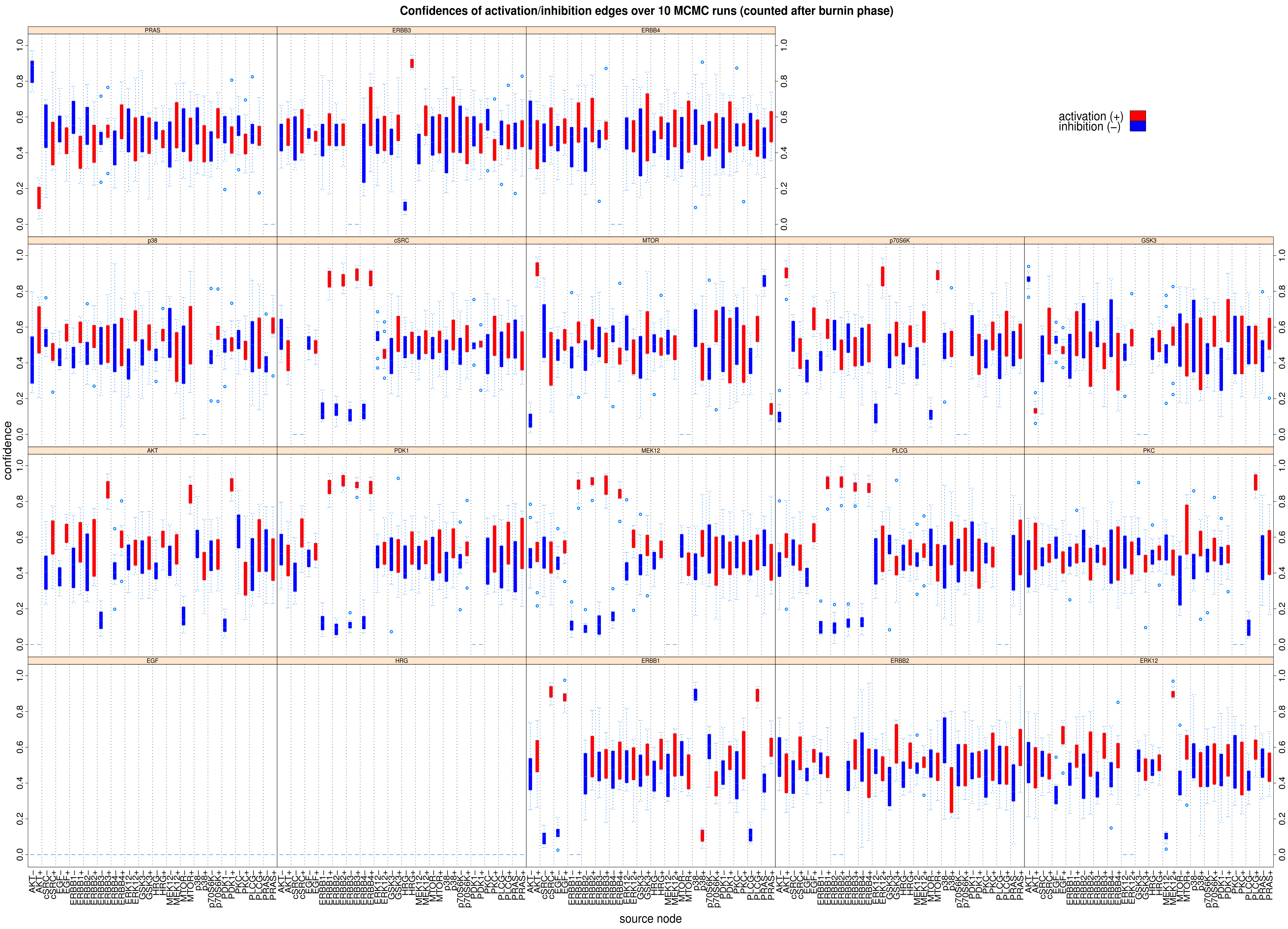

Suppl. figure S1: In each of the 10 inhibMCMC chains, activation and inhibition edges are sampled. The boxes show the frequencies of activations (marked in red) and inhibitions (marked in blue) to the total number of sampled edges (denoted as 'confidence', counted after the burn-in phase of 25000 iterations). Each panel represents the destination node of an edge. Consider the upper left panel 'PRAS'. In the first column, the blue box represents the confidence of the inhibition edge from AKT to PRAS, the red box represents the confidence of the activation from AKT to PRAS. If an edge is found as activation, its confidence represented as red box is significantly higher than the corresponding blue box (for the inhibition confidence). Note that self-activations and -inhibitions and edges to the stimuli nodes (EGF and HRG) were not allowed during inference, therefore there confidences are 0.
