# Supplementary material for: Inferring signalling networks from longitudinal data using sampling based approaches in the R-package 'ddepn'
Source: BMC Bioinformatics. 2011 Jul 19;12:291. doi: 10.1186/1471-2105-12-291 (PMC3146886; doi:10.1186/1471-2105-12-291)
Supplement: Additional file 2 — Model parameters for Gaussian distributions. Shows the Gaussian model parameters for active/passive states of each protein. [file 1471-2105-12-291-S2.PDF]

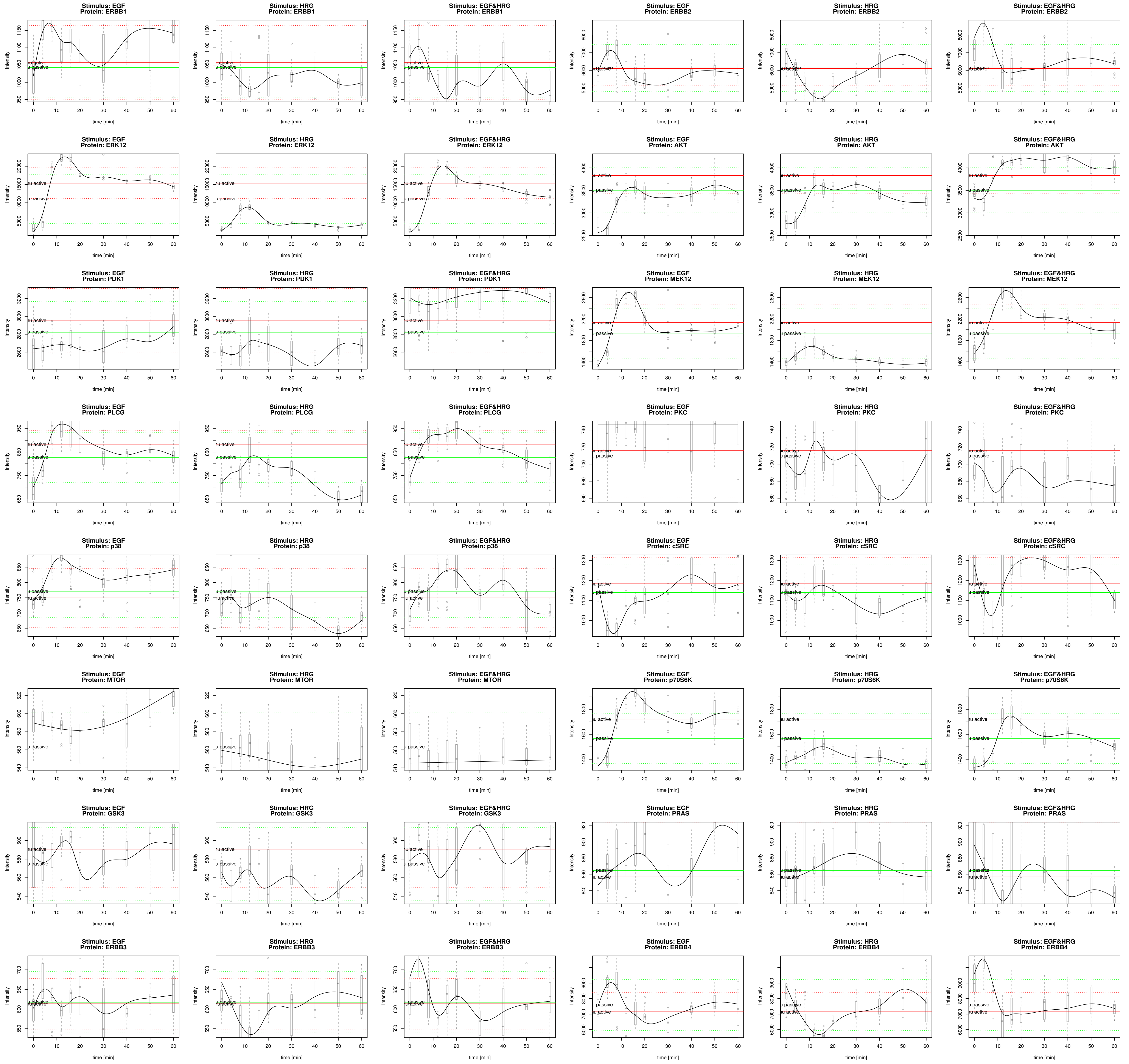

Suppl. figure S2: Antibody intensities for each stimulus EGF (Epidermal growth factor), HRG (Heregulin) and EGF+HRG, measured for each protein. The boxes show the distributions of the replicate protein intensities at each time point. A spline fit was performed for each protein and stimulus, shown as black continuous line. The red and green lines mark the fitted model parameters for the Gaussian distributions (solid: mean, dotted: standard deviation) for the active and passive state of the protein.
